# Leveraging Building Material as Part of the In‐Plane Robotic Kinematic System for Collective Construction

**DOI:** 10.1002/advs.202201524

**Published:** 2022-06-24

**Authors:** Samuel Leder, HyunGyu Kim, Ozgur Salih Oguz, Nicolas Kubail Kalousdian, Valentin Noah Hartmann, Achim Menges, Marc Toussaint, Metin Sitti

**Affiliations:** ^1^ Cluster of Excellence IntCDC: Integrative Computational Design and Construction for Architecture University of Stuttgart and Max Planck Institute for Intelligent Systems 70569 Stuttgart Germany; ^2^ Institute for Computational Design and Construction University of Stuttgart 70174 Stuttgart Germany; ^3^ Physical Intelligence Department Max Planck Institute for Intelligent Systems 70569 Stuttgart Germany; ^4^ Learning & Intelligent System Laboratory Technical University of Berlin 10623 Berlin Germany; ^5^ Computer Engineering Department Bilkent University Ankara 06800 Turkey; ^6^ Institute for Biomedical Engineering ETH Zurich Zurich 8092 Switzerland; ^7^ School of Medicine and College of Engineering Koç University Istanbul 34450 Turkey

**Keywords:** architecture, co‐design strategy, collective construction, construction robotics, task and motion planning

## Abstract

Although collective robotic construction systems are beginning to showcase how multi‐robot systems can contribute to building construction by efficiently building low‐cost, sustainable structures, the majority of research utilizes non‐structural or highly customized materials. A modular collective robotic construction system based on a robotic actuator, which leverages timber struts for the assembly of architectural artifacts as well as part of the robot body for locomotion is presented. The system is co‐designed for in‐plane assembly from an architectural, robotic, and computer science perspective in order to integrate the various hardware and software constraints into a single workflow. The system is tested using five representative physical scenarios. These proof‐of‐concept demonstrations showcase three tasks required for construction assembly: the ability of the system to locomote, dynamically change the topology of connecting robotic actuators and timber struts, and collaborate to transport timber struts. As such, the groundwork for a future autonomous collective robotic construction system that could address collective construction assembly and even further increase the flexibility of on‐site construction robots through its modularity is laid.

## Graphical abstract

In this article, a co‐designed collective robotic construction system based on robotic actuators that leverage timber struts as part of the kinematic system is investigated. A comprehensive workflow for deploying the system based on research from the robot hardware design, task and motion planning (TAMP), computational design and monitoring perspectives is realized to detail out the application of such a system to build structures in‐plane.

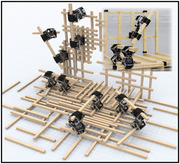

## 1 Introduction

Building construction is the biggest industry sector worldwide, but it faces considerable productivity and sustainability challenges. An integrative approach to design and construction allows these challenges to be addressed^[^ ^1^ ^]^. Recent interdisciplinary endeavors including the scientific fields of architecture, computer science, and robotics have showcased the promise of multi‐robot systems for more efficient and sustainable building construction.^[^ ^2^ ^]^ Collaboration of multiple robots has enabled automated construction processes to transition from the prefabrication of building elements or components in controlled, structured environments^[^ ^3^, ^4^, ^5^ ^]^ to the initial conceptualization of in situ robotic systems for on‐site construction.^[^ ^6^, ^7^, ^8^, ^9^, ^10^ ^]^ Modern construction sites are however generally characterized as unorganized with different activities occurring simultaneously and with deviations between digital models and physical conditions. Therefore, contemporary applications of robotics in the construction industry are primarily focused on the automation of conventional and at best slightly altered construction processes with industrial machines in controlled environments.

Contrary to this automation approach, research on collective robotic construction systems focuses on task‐ and site‐specific machines that are co‐designed with the architectural systems they construct. This research is beginning to prove that such systems can be more adaptive, scalable, and reusable with the ability to work in dynamic environments.^[^ ^11^, ^12^, ^13^, ^14^, ^15^, ^16^ ^]^ Current developments in collective robotic construction systems can be characterized by two major approaches, robots used as the building materials, in which the robots are part of the structure themselves, and robots used as a manipulator, in which they only serve to construct or deconstruct the structure. Although highly robust and reconfigurable,^[^ ^17^ ^]^ the former approach suffers from issues of power, complexity, and structural stability when scaled up, similar to challenges present in the field of modular robotics from which they draw inspiration.^[^ ^18^ ^]^ On the other hand, research in which the robot is a manipulator of the passive construction materials is currently demonstrating how multiple task‐specific robots can increase the complexity and efficiency of construction.^[^ ^19^, ^20^, ^21^, ^22^, ^23^ ^]^ This has led to the construction of building artifacts that are much larger than the machines themselves. Although they are becoming more and more sophisticated, none of these systems can build architectural artifacts with materials that exist in the construction industry, thus limiting their practical applications.

**Figure 1:**
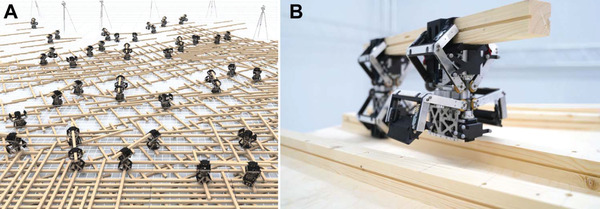
Collective robotic construction system that leverages the building materials for construction as well as part of the robotic kinematic system. A) Digital representation of the proposed collective robotic construction system, composed of timber struts and robotic actuators, which combine to form modular robot‐material kinematic chains that can reconfigure throughout the construction process. B) Photo of two robotic actuators forming a kinematic chain with one timber strut. Here, the size of the robotic actuator prototype is 138 (depth) × 179 (width) × 309.5 (height) mm^3^ when both grippers are closed.

This work combines these two approaches to a collective robotic construction system into a system that leverages the building materials for construction as well as a part of the body of robotic hardware for locomotion and material manipulation (**Figure** **1**). The system is composed of timber struts and robotic actuators, which combine to form modular robot‐material kinematic chains that can reconfigure throughout the construction process. Kinematic chains are similar to industrial robotic arms composed of links and joints but can break apart or rearrange during the construction process to form chains with varying degrees of freedom (DOF). Given specific goals during the construction process, high‐level decisions on task sequence and motion path plans are autonomously computed by a custom planner and then executed by the robotic actuators to build architectural artifacts. Timber struts and active robotic actuators can be added to the construction system i) to execute more complex tasks, which require higher DOF, ii) to achieve geometric variation, or iii) to change the built artifact during its assembly. This allows for the increased flexibility of the overall system while maintaining a minimal number of actuators.

The success of our system relies on the integration of expertise from various fields including robotics, computer science, and architecture. The research contributions are the development of robotic hardware for in‐plane assembly, task and motion planning (TAMP), and the computational design of building artifacts. As such, this paper is built upon the collective robotic construction system in^[^ ^23^ ^]^ through the co‐development of specific methods into a single workflow for deploying such a system. Specifically, we further analytically i) analyzed and developed the robotic hardware, including major updates to the gripping mechanism, ii) introduced methods for designing architectural affects to be built with the system, iii) established an advanced approach for task and motion planning, and iv) integrated a centralized perception system for error correction, all of which are integrated into the workflow.

The collective construction system of timber struts and active robotic actuators is evaluated with a series of experiments as well as validated with five hardware demonstrations, showing three construction‐related tasks. These tasks are inherent to working with the proposed system for planar constructions and include: locomotion, dynamic kinematic chaining, and transportation. By providing an example of how a task‐specific robot can be designed to leverage real building material, we begin to lay the groundwork for an autonomous collective robotic construction system that can address real construction assembly. The system has the capacity to not only expand the existing automation in collective construction practices through the use of real‐world building materials but also further increase the flexibility of on‐site construction robots through its modularity.

## 2 Results

### 2.1 Rotational Axis Design for Modularity of the Robotic Actuator in Kinematic Chains

**Figure 2:**
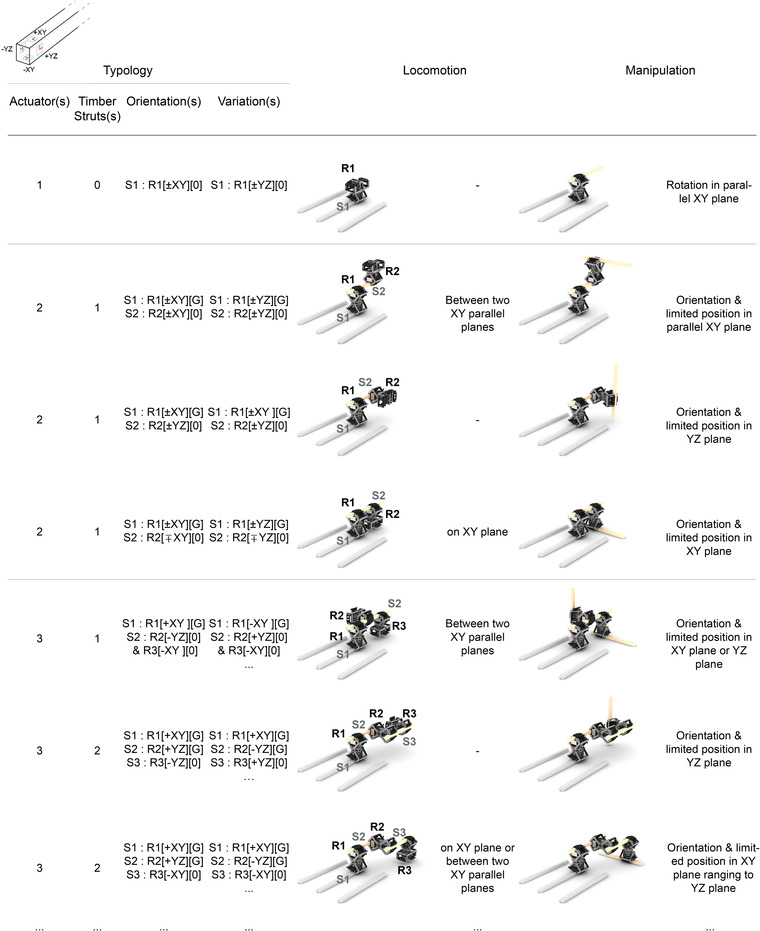
Matrix of the kinematic chain typologies and their locomotion and manipulation abilities utilized in order to understand and analyze the capabilities of the proposed collective robotic system. Typology defines the overall connectivity of the robotic actuator(s) and timber strut(s) in a kinematic chain. Specifically, the orientation of the robotic actuators (R1, R2, R3) are references to the planes of the struts (S1, S2, S3) as described in the upper right image and labeled in the locomotion images. The struts on the bottom left in grey color are fixed to the environment. Variations are typologies with the same capabilities of locomotion and manipulation. Locomotion explains the environment in which the specified kinematic chain could move on its own. Manipulation describes how the kinematic chain could manipulate an additional strut if gripped at the end of the kinematic chain, both with an image and text description.

The main objective here is to co‐design a modular collective robotic construction system in which timber struts are assembled by simple robotic agents. The system developed is based on the interaction between passive timber struts and active single‐axis robotic actuators. Since a single robotic actuator is unable to move on its own, the combination of interactions between the robotic actuators and timber struts in forming, breaking, and adapting kinematic chains allows for the locomotion of kinematic chains and manipulation of timber struts (**Figure** **2**). In order to allow for different capabilities, the configuration of the kinematic chains in the assembly process can be dynamic. This means that the location of a robotic actuator in a kinematic chain can vary throughout the construction process. As such, the rotational axis of the actuator needs to be able to occupy any position within the kinematic chain. Therefore, the robotic actuator must be designed for the worst loading condition, which occurs when an actuator is required to rotate the entire kinematic chain. When a kinematic chain has multiple actuators, dynamic load from all the actuators in the chain must be considered. Thus, in designing the system, kinematic chains are optimized for a minimum number of actuators.

### 2.2 System Design for In‐Plane Construction

The necessary requirements on and general complexity of the construction system increases with the number of actuators permitted within a single kinematic chain. However, kinematic chains consisting of a maximum of two robotic actuators are enough in order to showcase the ability of our system to autonomously perform assembly tasks necessary to build architectural artifacts. Collaboration of kinematic chains of such complexity would allow the assembly of struts into planar architectural artifacts.

**Figure 3:**
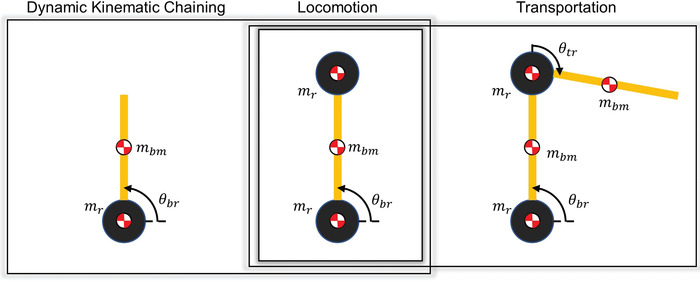
Loading conditions for the kinematic chains in each of the three assembly tasks. Locomotion contains one type of kinematic chain (2 robotic actuators and 1 timber strut) while dynamic kinematic chaining and transportation contain that of locomotion plus an additional type of kinematic chain.

The assembly of artifacts in plane can be achieved with three assembly tasks: locomotion, dynamic kinematic chaining, and transportation, which all involve the rearrangement of both robotic actuators and building material. These tasks incorporate two, three, and four robotic actuators respectively. Each has different load cases on the robotic actuators. **Figure** **3** describes the load cases that occur in these three tasks.

Of the three tasks involved in the assembly of planar architectural artifacts, transportation in the vertical plane would require a single actuator to withstand the largest load. The required actuator torque in this loading case is given as:

(1) $$\tau_{\mathrm{required},\mathrm{ro}}=I\ddot{\alpha }+C_{e}\omega^{2}+C_{o}\omega +G$$

where *I* is an inertia matrix, *α* and *ω* are angular acceleration and velocity matrixes of the rotating actuators, respectively, is the matrix of the centrifugal forces, *C_o_* is the matrix of the Coriolis forces, and *G* is the matrix of gravitational forces. Generated dynamic torques from the motion of a kinematic chain, which are related to the angular acceleration and velocity, occupy a smaller portion than gravity due to the low acceleration and velocity. Therefore, inertial, centrifugal and Coriolis forces are not considered. The maximum static load occurs when the kinematic chain is fully stretched against gravity in the vertical plane (*θ*_*br*_ = 0°, *θ*_*tr*_ = 0°). Therefore, in order to calculate the maximum static required torque for the actuator, Equation 1 becomes:

(2) $$\tau_{\mathrm{required},\mathrm{ro}}=\frac{gl_{\mathrm{bm}}}{\eta_{\mathrm{ro}}n_{\mathrm{ro}}}\left(\sum_{i=1}^{n_{r}}2\left(i-1\right)m_{r}+\sum_{i=1}^{n_{\mathrm{bm}}}2\left(i-1\right)m_{\mathrm{bm}}\right)$$

where *n_r_* and *n_bm_* are the number of robots and struts in the kinematic chain, respectively, *g* is the gravitational acceleration, *l_bm_* is the length of the building material, *η*_*ro*_ is the efficiency of the selected motor, is the net gear ratio in the rotating mechanism, and *m_r_* and *m_bm_* are the mass of the robotic actuator and building material, respectively.

### 2.3 Performance Study on Kinematic Chains

A series of experiments were conducted in order to characterize the performance of the kinematic chains generated by two robotic actuators. These experiments were conducted with varying lengths between actuators in order to characterize the prospective performance of the kinematic chains with varied typology. The characterized results are used as factors to plan assembly with the proposed system, such as in designing the architectural artifact to be assembled and planning the assembly process. System performance is different when the main axis of a robotic actuator is oriented parallel or perpendicular to gravity, and thus performance was tested in both horizontal and vertical planes.

**Figure 4:**
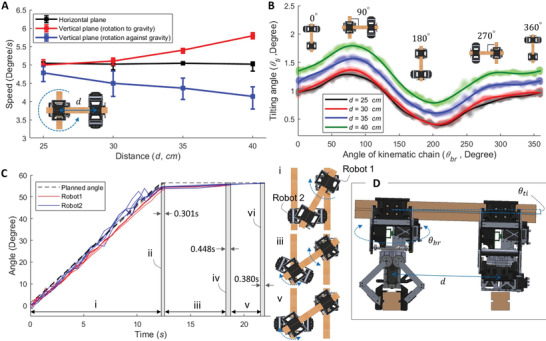
Experimental performance of the system for in‐plane horizontal and vertical motion of two robotic actuators. A) Robot actuator rotation speeds during locomotion in the horizontal and vertical planes. Results were measured and averaged based on ten trials. B) Tilting behavior of the kinematic chain during rotation. The shaded lines denote the measured raw data from ten trials, and the solid lines denote averaged results from the ten trials. C) Correction performance results for orientation and position after a rotation: i) executing the planned angles, ii) end of executing, iii) correction of the position and orientation for the robotic actuators 1 and 2, iv) end of correction for the robotic actuator 1, v) correction of the position and orientation for the robotic actuator 2, vi) end of correction for the robotic actuator 2. The results were from five trials. D) Schematic view for the tiling issue of the kinematic chain.

The measured robotic actuator rotation speed (**Figure** **4**A) is the speed at which one robotic actuator rotates the entire kinematic chain. The rotational speed in the experiments was determined by the rotation motor and related gear connections. However, friction in the mechanisms of a single robotic actuator or related to the general assembly of the kinematic chain could influence this speed. Therefore, we measured the rotation speed through a series of tests at different distances between the robotic actuators. In the horizontal plane, the speed with different distances between the robotic actuators was the same. In the case of the locomotion in the vertical plane, the rotation speed varied. The speed increased with longer distances between robotic actuators when they rotated down with the direction of gravity, while the speed decreased when they rotated up against gravity. This was due to the extra force from the weight of the kinematic chain. Rotational speed was utilized to estimate the overall operation time of an assembly task.

In the horizontal plane, gravity does affect the formation of stable kinematic chains. Due to the variance in the machining of both the timber struts and parts of the actuator body as well as the general hardware assembly, small tolerances in the formation of kinematic chains could cause tilting of a kinematic chain from the robotic actuator that rotates it (Figure 4D). Tilting could cause the completion of an assembly task to fail. Further experiments were conducted to characterize this issue. Specifically, tilting angles were measured at different spacing between robotic actuators and angles of the kinematic chain. Figure 4B confirms that when kinematic chains were parallel to the strut to that they grasped, tilting was less due to the geometry of the gripper. Variation in similar loading conditions occurred due to tolerances directly from the milling of the timber. Correlating the information between the distance of tilting and the length between robotic actuators determined how far a kinematic chain could safely reach. This served as a direct input for the system design methods, which determined what design artifacts can be assembled by the system.

Finally, correction of the position and orientation of the robotic actuators in a kinematic chain is important to the success of any gripping activity and was analyzed in further experiments (Figure 4C). It furthermore affects the overall time of an assembly task and needs to be accounted for when estimating operation time. Correction occurs when gripping happens in the planned motion. When correcting, the system uses the measured positions of the robotic actuators from the external visual tracking system to correct their position and orientation (Figure 4C, iii and v). A bang‐bang controller was implemented for this correction. The bang‐bang controller was designed to run until the average angle error between the planned and actual angles was under 0.3°, which was enough for successful gripping.

### 2.4 Physical Demonstrations

**Figure 5:**
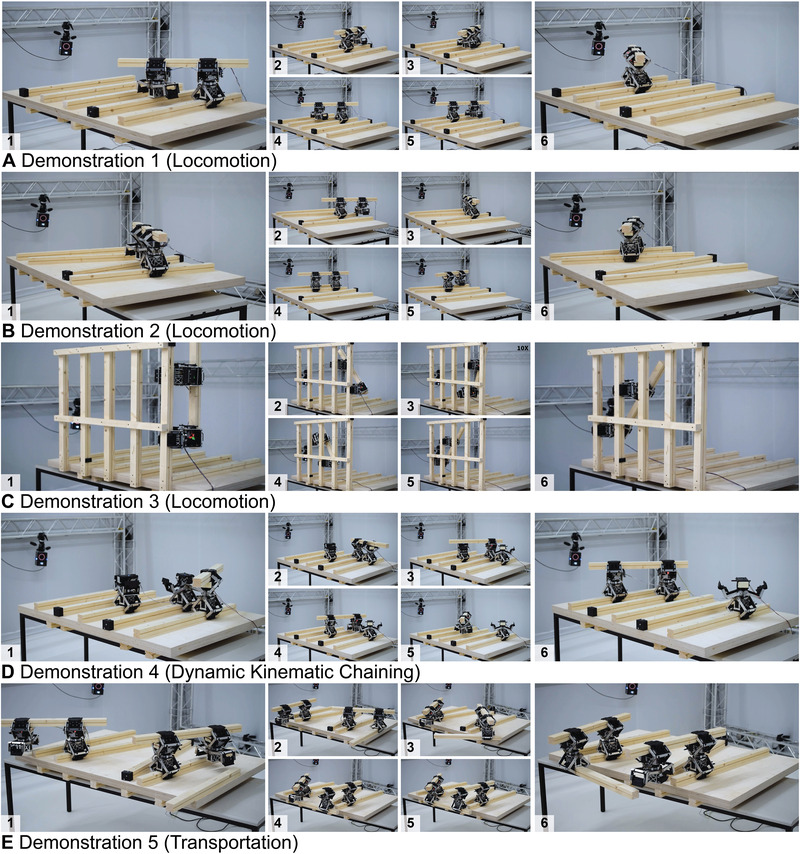
Photo sequences for five physical demonstrations (Demonstration I – V). 1) At the start of the demonstrations, the robots and struts were placed in the configurations seen in the far left and 6) navigated to the positions seen on the far right. A) Demonstration I: Locomotion in ground‐parallel regular environment with two robotic actuators. B) Demonstration II: Locomotion in ground‐parallel random environment with two robotic actuators. C) Demonstration III: Locomotion in ground‐perpendicular regularized environment with two robotic actuators. D) Demonstration IV: Dynamic kinematic chaining with three robotic actuators in a regular environment. E) Demonstration V: Transportation with four robotic actuators in a random environment.

In order to showcase the three assembly tasks (locomotion, dynamic kinematic chaining, and transportation) for in‐plane construction, five physical demonstrations were designed, planned, and executed. The demonstrations consider all robotic, material, architectural, and computational constraints of the developed system as well as the results of the performance study discussed in the previous section. In the demonstrations, each task was specified at a high level as a configuration of robots, struts, and goals to a combined task and motion planner, which generated instructions for the robotic actuators to execute. The execution was monitored by a centralized visual tracking system accompanied by an algorithm to adjust for tolerances between the planned motions and the real motion of the physical system. **Figure** **5** shows snapshots from each of the demonstrations. Videos of all demonstrations are given in Movie S1 (Supporting Information). The demonstrations were run 10 times each in order to validate robustness and reproducibility.

In Demonstration I–III, a kinematic chain composed of two robotic actuators and one timber strut performed the task of locomotion. Locomotion is the action of bringing a kinematic chain from one location to another. The construction environment of each of the three demonstrations further contained four or five fixed timber struts, which provide the structure for the kinematic chains to locomote. For Demonstration I and III, the fixed struts were spaced equally apart either parallel to the ground (Demonstration I) or perpendicular to the ground (Demonstration III). For Demonstration II, the struts were positioned with varied distances and angles parallel to the ground as seen in Figure 5B. These three demonstrations displayed the ability of the kinematic chains to locomote in the horizontal and vertical planes with varied environment configurations.

**Figure 6:**
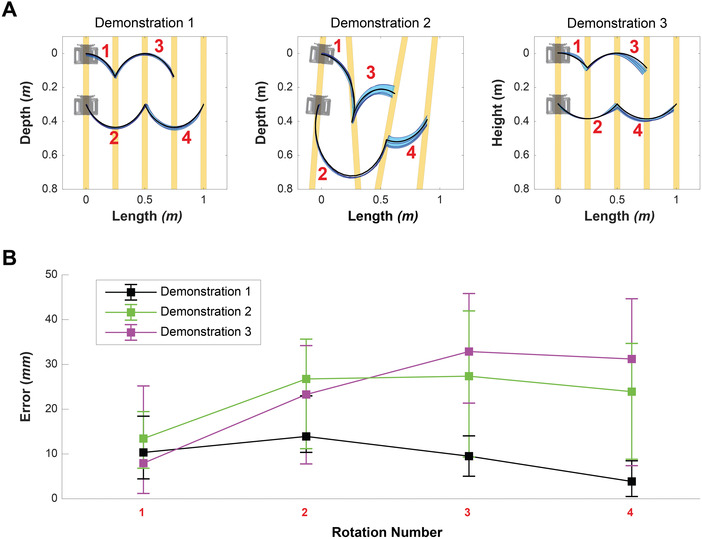
Performance results of the locomotion task demonstrations. A) Trajectory comparison for the Demonstration I, II, and III. The red numbers denote the order of rotations required to successfully complete the demonstration. The solid black lines denote the planned trajectories for each robotic actuator. The light blue volume together with the dark blue lines denote the range and exact measured trajectories of the robotic actuators during the ten trials of each demonstration. B) Error between the points of planned and measured trajectories at the end of each rotation. The rotation numbers in the *x*‐axis relate to red numbered rotations in the upper graphs.

**Figure** **6** shows comparisons between the planned and actual measured trajectories during the locomotion task in Demonstration I‐III. Demonstration I shows the basic performance of locomotion, as the environment in which the kinematic chain locomoted has an equal spacing of parallel timber struts. For this reason, the rotations were continuously repeated and the error of the actual locomotion to planned trajectories was relatively smaller than other demonstrations (Figure 6B). In addition, the error was not significantly changed at each rotation. On the other hand, Demonstration II had irregular fixed struts, which introduced variance into the system (Figure 6B). Demonstration III revealed that gravity in the vertical plane caused the kinematic chain to slip lower as they completed the task of locomotion. As a result, the errors were also higher than Demonstration I.

Demonstration IV built upon locomotion by introducing the dynamic nature of the system that is inherent to its modularity. In this demonstration, three robotic actuators were gripped to struts in the same environment as in Demonstration I. Three robotic actuators worked together with one additional timber strut to move two robots to a specific location. The planned sequence, as depicted in Figure 5D, involved the first actuator passing a strut to the second robot (Figures 5D(1,2)). The second actuator rotated this strut in order to pick up the third robot (Figure 5D(3)). The kinematic chain between the second and third actuator is then locomoted to the goal location to fulfill the task (Figure 5D(4–6)). This demonstration proved the ability of the system to form different kinematic chains throughout an assembly process. It further demonstrated how robotic actuators could be used to rearrange kinematic chains to perform different actions, for example changing the length between two robots in a kinematic chain to have a different reach length.

Demonstration V involved the transportation and placement of timber struts. In the same environment as in Demonstration II, four robots and two timber struts collaborated to transport a strut from one location to another. In Demonstration V (see Movie S1, Supporting Information)), an agent‐based model (ABM) was used to design the final artifact, specifically indicating the goal location of the transported strut. The final demonstration verified how the system could be utilized to assemble struts by moving them to assembly locations chosen while in the process of construction.

## 3 Discussion

We present a modular collective robotic construction system in which conventional building material forms a part of the robotic kinematic system, therefore blurring the lines between material, robot, and building artifact. Hardware demonstrations proved that physically prototyped robotic actuators can autonomously grip onto timber struts. Through gripping and releasing the timber struts, robotic actuators can form stable kinematic chains to increase or decrease their DOF, which in turn affect their capability to move in space and perform manipulation tasks of varying complexity. This shift in the ability of the system as altered by the number of active robotic actuators is evident from the demonstrations, which transition from two robotic actuators locomoting in their environment to four robotic actuators positioning timber struts for assembly. In the demonstrations, kinematic chains of fixed or dynamic typologies worked both separately and together in order to move robotic actuators and timber struts in space. The tasks presented in the demonstrations comprise a set of assembly primitives that would allow for the system to build architectural artifacts.

**Figure 7:**
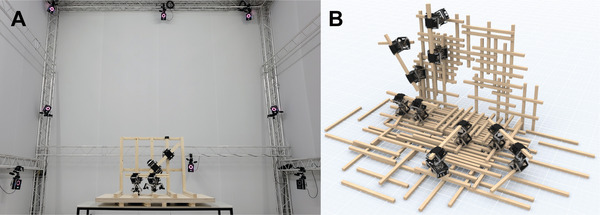
Proof‐of‐concept 3D construction experimental and simulated demonstration with the proposed system. A) Four robotic actuators were externally lifted between the various planes, which could be built autonomously using the position and orientation feedback from the external visual tracking system. B) Digital simulated representation with ten robotic actuators working on the same architectural artefact.

The five demonstrations further showcase the proposed system working in two different planes with regular or irregular strut arrangements. Considering the kinematic chains are in their worst loading condition in the vertical plane, this proves the ability of the system to operate in a large range of designed and more random configurations regardless of the direction in which the robotic actuators are required to work against gravity. The proposed system can assemble architectural artifacts in a plane with varying degrees of density and orientation, in which decisions on the exact distance between struts or their angle to each other can be made in response to the current status of the constructed artifact. This ability of the system to work in 2D planes is a direct result of the hardware of the robotic actuator, which has a carrying capacity of one other robotic actuator and two timber struts. 3D architectural artifacts are currently achievable by externally lifting the robotic actuators into new planes. The combination of planar assemblies can result in the formation of architectural spaces (**Figure** **7**). Further research on the hardware is required in order to transition to the assembly of freeform 3D artifacts.

Several challenges were observed in the experiments and demonstrations that would need to be overcome in future work to improve the robustness of the system. Although our external visual tracking system is able to correct for alignment issues and iteratively planning for overall deviations, failures in the various demonstrations were observed due to machining tolerances, as timber is a heterogeneous material with mechanical properties that can change along its cross section. Although manual adjustment allowed for the completion of all the demonstrations, further advancements in the gripper to allow for more tolerances, and further stiffening the body of the robotic actuator would help the autonomy of the system. Local sensing techniques could also help guarantee continuous stable gripped connections between the timber struts and robotic actuators throughout the construction process. Updates to the algorithm, which actuates the gripping according to the measurements of torque from the gripping actuators or other additional sensors could help increase the system robustness.^[^ ^24^ ^]^ Sensor feedback loops combining both local and global sensing would further allow for the creation of material and environment‐aware open‐ended building artifacts to be assembled with the system.

Another ability of the system that would be required for real‐world construction applications would be the joining of timber struts together. Thus, future work would involve developing an end‐effector for the robotic actuator. An end‐effector, such as a drill with an automatic screw feeder, can be connected to either side of the robotic actuator, parallel to its axis in order to give the robotic actuator the ability to connect the struts that it grips with other struts or wood in its environment. The exact development of the end‐effector will draw from existing explorations on end‐effectors designed for modular robotic systems^[^ ^18^ ^]^ and the effector introduced in.^[^ ^23^ ^]^

## 4 Conclusion

Collective robotic systems like the one proposed here have a number of advantages, especially as compared to existing industrial‐scale robotic systems. First, they can work on unlimited scales because they are based on mobile machines, thus their movement range is theoretically unrestricted. Next, the machines must collaborate, which allows for accomplishing tasks of varying complexity. The machines may further work in parallel fulfilling multiple tasks at the same time. Moreover, they are lower in cost than systems with industrial machines which require large setup areas with special power as well as anchoring requirements. Finally, they are robust to failure. If a single machine is damaged or destroyed, others can take its place. We believe that collective robotic systems have the potential to revolutionize robotic architectural construction, in which these advantages could play a vital role in dealing with the unstructured nature of construction. We therefore further co‐designed a collective robotic system that leverages real building material, from the perspectives of robot hardware design, task and motion planning (TAMP), and computational design of building artifacts, in order to showcase how such systems can be deployed for real architectural construction.

## 5 Experimental Section

**Figure 8:**
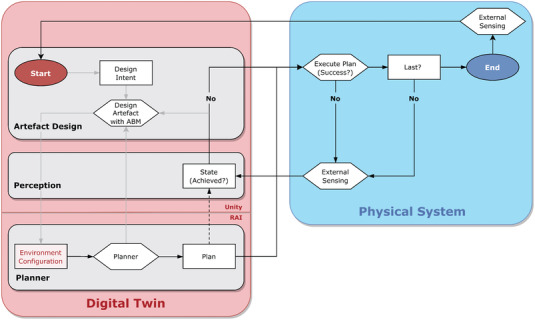
System architecture flowchart. Strategy for the exchange of information between the digital and physical methods developed for the completion of construction tasks. The digital methods include those for artefact design, perception and task and motion planning (TAMP). Planned robotic motions, derived from designs created with the ABM, are compared to the physical state of the system in order to successfully execute construction tasks.

The following section describes the various components developed in order to deploy the proposed collective robotic construction system. **Figure** **8** is a flowchart that visualizes the exchange of information between our co‐designed methods. The general workflow involved constant feedback between i) a digital twin, which includes methods for artifact design, perception, and planning, and ii) the physical system, composed of the timber struts, robotic actuators, and an external visual tracking system. The process of assembly started with a designer defining design intent using our ABM, the generative design tool. The ABM then iteratively provides the planner with environment configurations to be solved that include information on the current locations of robotic actuators and timber struts as well as a task to be achieved. Plans, which include action sequences for the robotic actuators, were generated by the planner and then sent to the physical system for execution. This process was monitored and compared with the planned motions in order to adjust for tolerances and ensure the successful execution of assembly sequences. Once a plan was finished, the digital twin is updated with the final state of the physical system. By iteratively sending the artifact designs to the planner, any deviation between the measured location of the robotic actuators and the timber struts could be accounted for in subsequent steps of the assembly. As the Demonstrations I–IV did not involve the placement of new timber struts in the environment, the planner was provided with hand‐designed configurations to be solved. The specifics of each method are defined in the following sections.

### Timber Strut

The building material, an otherwise completely passive element in the robotic kinematic system, had to fulfill two major criteria in order to be leveraged by the robotic actuators. First, it needs to support the weight of the robotic actuators while still being light enough to be transported by them. Second, it needs to facilitate stiff gripped connections with the robotic actuators to allow for the stable formation of kinematic chains. Therefore, lightweight timber struts with square cross section of 50 mm by 50 mm as the building material was chosen. The struts were made from spruce, a standard softwood used for lumber, which allowed sawn tolerances of 1 mm.^[^ ^25^ ^]^ The length of the timber struts was dependent on the application or the designed scenario in which it was utilized. However, in order to guarantee that two robots could grip onto a timber strut at the same time to form a kinematic chain, the length of the strut must be at a minimum of 400 mm.

### Robotic Actuator

**Figure 9:**
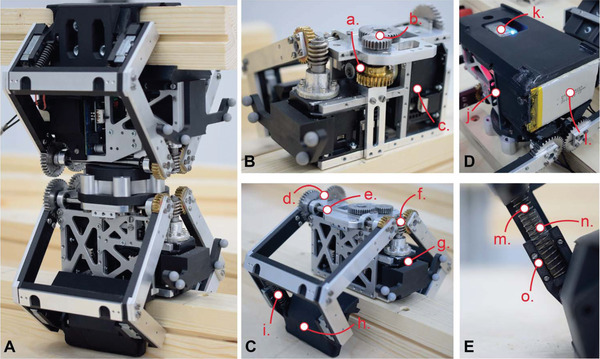
Photos of the various components of the robotic actuator. A) Fully assembled robotic actuator. B) Rotating mechanism [ i) worm gear assembly (1:25), ii) spur gear connection (26:40), iii) rotation motor]. C) Gripper mechanism [ iv) spur gear assembly (1:1), v) Gripper arm shaft, vi) worm gear assembly (1:25), vii) gripper motor, viii) gripper paddle, ix) lifting mechanism]. D) Electronic parts [ x) controller (OpenCM 9.04 with 485EXP, ROBOTIS), xx) Bluetooth module (BT‐410, ROBOTIS), xl) battery]. E) Lifting mechanism [ l) spring, xc) linear rail, c) moving part on the linear rail].

The design concept for the robotic actuator was to perform as an axis like that of an industrial robotic manipulator. However instead of having permanent links on either side of the axis, which would further connect to other axes, the actuator was equipped with two grippers, the upper gripper and lower gripper on either side of its single axis. In addition to the mechanism required for gripping (**Figure** **9**C), each gripper is equipped with further parts that enable the robotic actuator to rotate, grip and lift building material and communicate with the digital twin. Lifting the timber struts is further introduced from the previous designs in order to avoid any friction during transportation.^[^ ^23^ ^]^ The lower gripper contains the mechanism for rotation (Figure 9B), and the upper body contains the major electronics including the microcontroller and Bluetooth module and battery (Figure 9D). Calculations for the battery can be found in the Supplementary Information.

**Figure 10:**
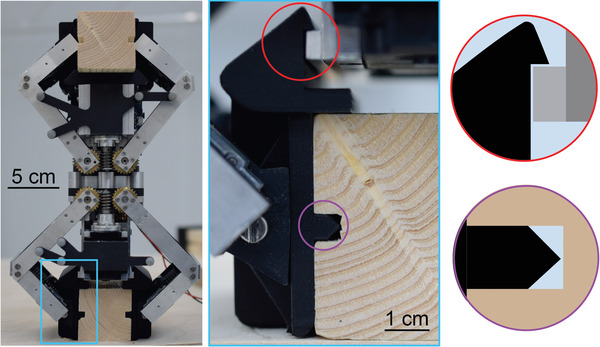
Passive mechanisms integrated into the robotic actuator. Zoomed‐out and close‐up (indicated as the blue rectangle) photos of the robotic actuator gripping a timber strut. The red circles denote the stiffening mechanism and the purple circles represent the alignment mechanism.

The two grippers allowed the robotic actuators to join with the timber struts to form kinematic chains. Kinematic chain formation allows for more connected DOF in the system and gives the robotic actuators the ability to perform assembly tasks. The formation and motion of kinematic chains were dependent on stable connections between the timber struts and the gripper of the robotic actuator. This system requirement translates physically to the integration of two passive mechanisms as upgrades to the robotic hardware from,^[^ ^23^ ^]^ which lock the gripper of the robotic actuator to the timber struts when the gripper was closed. The two mechanisms, i) an alignment mechanism, and ii) a stiffening mechanism, enable the robotic actuator to make consistent, stable connections to the timber struts (**Figure** **10**).

The development of the alignment mechanism involved the co‐design of the gripper and what it grips, an approach being utilized in modular robotics^[^ ^26^ ^]^ as well as other collective robotic construction systems.^[^ ^14^ ^]^ Specifically, a tongue and groove type joint was designed between the gripper paddle and timber strut. A square groove was milled into the timber struts, which matches to the geometry of the paddles of the gripper of the robotic actuator. A square groove was chosen as it would require the least amount of further processing. It was therefore the most‐cost effective option, when considering the already rectilinear timber struts. The symmetry of a square was further important to the co‐design between the timber strut and robotic actuator as it would allow the struts to be gripped from any side. This simplifies both the design and planning methods in that any strut could be used anywhere in the system. The tongue geometry of the gripper paddle, which was square with an angled tip, helps the gripper align to a timber strut that it aims to grip at the correct position and orientation.

The stiffening mechanism was a clipping mechanism between the paddle geometry and the frame of the body of the robotic actuator. This allows for further stabilization of the robotic actuator while gripping, specifically against forces perpendicular to the timber strut while it was gripping. Together the two mechanisms lock the robotic actuator to the timber strut when the gripper was closed.

Each gripper can further grasp, lift and release timber struts by opening and closing the gripper arms. The calculation of forces for gripping in different states, as discussed in the Supplementary Information, was conducted in order to select an appropriate motor for the grippers. The body of each gripper was made of milled aluminum frames which hold the motors: in the upper gripper one motor and the lower gripper two motors (one for gripping and one for rotation). The frames further support the shafts, manufactured from titanium, on which the gripper arms and paddles are mounted. The paddles as well as some other subsidiary parts were made from micro carbon fiber filled nylon printed by a Markforged Onyx Pro (Gen 2) 3D printer. This includes a mount for markers, which could be detected by the Vicon cameras from the external visual tracking system. Further, detailed specifications of the robotic actuator are listed in Table S4 (Supporting Information).

### External Visual Tracking System

The external vision system was composed of 16 Vicon Vantage+ cameras, which read location and rotation data of markers located on the robotic actuators and timber struts. This information was streamed to a central computer at 200 Hz.

### Digital Twin

**Figure 11:**
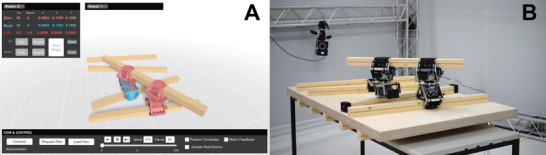
User interface for executing and monitoring the robotic construction system. A) Digital twin with ability to interface with the planner, run the ABM, execute plans, and monitor the physical system. B) Status of the physical system as represented in the digital twin.

The digital twin serves to design, plan, communicate instructions, and monitor the construction process. A custom interface was developed using the Unity Game Engine, which allows for both manual and autonomous running of the system (**Figure** **11**).

### Artifact Design

In architectural design practice, top‐down processes are commonly utilized in which post‐rationalizations of design were made after initial design conception in order to reflect fabrication and construction constraints. When building with robotic construction systems, where machines fabricate an architectural artifact, the opportunity arises for bottom‐up design methods to be utilized in which the design of a built artifact emerges from the understanding of all the system parameters.^[^ ^27^ ^]^ The fabrication as well as material system constraints could be used to create generative tools for designing artefacts assembled by collective robotic systems.

To take advantage of this possibility of integrating more building‐ and automation‐specific information into the design process with bottom‐up methods, an ABM for design was developed. ABMs study how a large number of autonomous elements could self‐organize through the definition of both individual and collective behaviors, which were designed in order to reflect the system they represent. Our ABM explores this self‐organization for the design of architectural artifacts with behaviors explicitly designed based on our proposed system. With our ABM, the designer therefore did not have to consider all of the details of the system but rather adjusts design intent parameters of boundary region, timber strut supply location(s), density, orientation, and placement priority in order to design the artifact built with the system. By tuning these parameters, which were stored in the agent environment during or prior to the assembly, the built artifact and assembly process was affected as discussed in **Table** **1**. As compared to existing ‘blueprint’ or predefined design approaches with collective robotic systems,^[^ ^6^, ^12^ ^]^ the designer was no longer required to be an expert of the system in order to design the artifact to be assembled.

**Table 1:** Effect of the design intent parameters on the overall designed artefact and assembly process

Design Intent Parameter	Digital Representation	Effect on overall designed artefact and assembly process
Boundary Area	Volume	Defines the general working area
Timber Strut Supply Location(s)	Curve(s)	Defines the starting location(s) of assembly
Density	Scalar Field	Controls how close together timber struts can be placed
Orientation	Vector Field	Controls the relative angle of timber struts
Placement Priority	Scalar Field	Determines where regions of work area are more important for placement of timber strut

**Figure 12:**
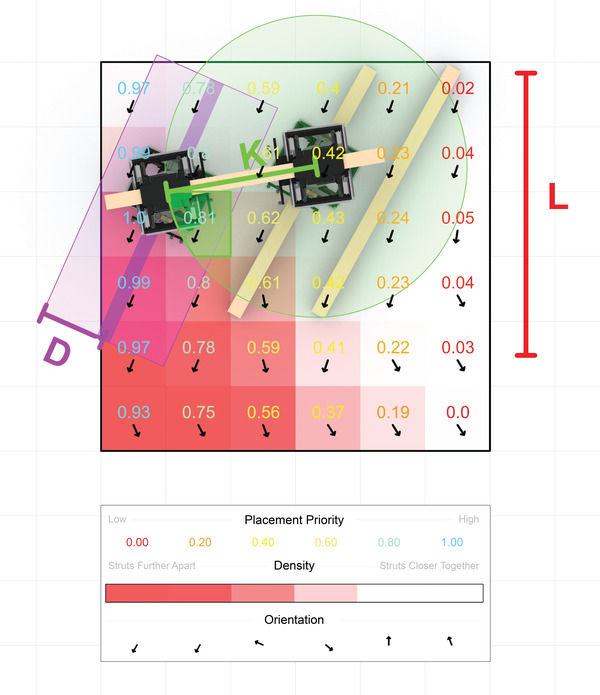
Visualization of the design intent parameters utilized in the ABM. At each iteration of the model the design intent parameters determine the next strut to be placed (shown in purple color) and pose of the agent or kinematic chain that would place it. L is the supply location where timber struts could be fed into the boundary area. Placement priority is a scalar field, which specifies the importance of building within the boundary area. The location with the highest placement priority is determined within a sight region (K) of the agent (shown by the green circle). Density is a scalar field that describes how far other already placed struts should be away from the new one (D). Red in the density field means that struts should be placed further apart from each other. Orientation designates with a vector field the orientation on struts. The behaviors of the agents in the ABM utilize these three fields in order to determine the location of the next strut to be placed. Variation can occur from the exact values of the three fields by adjusting an acceptable range of tolerance from the defined values.

Boundary area was defined as a volume, which might contain voids, and influences the overall size and shape of the construction. Density and orientation were defined as a scalar field and vector field respectively and influence the general compactness of the struts and their angle of placement. Although orientation is completely free, density was restricted by the hardware as the grippers need the space to return to the fully retracted state and release from struts. Timber strut supply locations and placement priority had less effect on the overall design but rather influence the assembly process, which the former specifically described as a curve to represent where timber struts were fed into the system and the latter as a scalar field. The dimensions of each of the fields were the same and contained within the boundary area. The design intent parameters were further depicted in **Figure** **12**.

In the developed design tool, each agent represents an abstraction of a kinematic chain. At each iteration of the ABM, each agent proposes an end pose for the kinematic chain it represents as well as a possible location for the next strut to be fixed into the environment (Figure 12). This location was not only informed by the design intent parameters set by the designer but further by the distance between the robotic actuators in the kinematic chain that places it. Therefore, the designer must define the agents by defining the kinematic chains and thereby the evaluated reach, as a result of the tilting performance study, along with the design intent parameters in order to run the ABM.

The agents had three sequential behaviors, seek, orient, and cull, which run at each iteration of the ABM. The seek behavior defines the direction of the next placement location based on the placement priority parameters. A sight parameter for the agents was introduced for this behavior, which was not represented in the physical system but allows us to define the ability of each agent to read the design intent field parameters from the environment. The orient behavior defines the angle of the next placement location based on the orientation parameters. The cull behavior adjusts the placement priority parameters in the environment based on the density parameters. At each iteration of the model, the behaviors run sequentially and return a goal location for a strut as well as the final pose of the kinematic chain. This information was then compiled along with the other struts that exist in the environment as a JavaScript object notation (JSON) file, which was then sent to the planner whose role was to solve for a task sequence and motion path that results in the intended design. This iterative process had further advantages as it allows for the designer to be integrated in the process of assembly, making them able to make changes of the design artefact on the fly. This juxtaposes top‐down methods in which the designer must decide on the entire design before the assembly process begins

### Task and Motion Planning (TAMP)

TAMP was concerned with i) finding high‐level action sequences, and ii) finding suitable motions to execute the action sequences, that satisfy both the logical and physical goals of the task. In this work, a planner was devised that extends Logic‐Geometric Programming (LGP)^[^ ^28^ ^]^ to solve the TAMP problem imposed by the construction related tasks as explained in the previous sections. A core extension was to allow for kinematic switches during the plan that alter the topology of the overall system's kinematic tree, which poses a significant challenge as the existing planner consider fixed typology robots. More, specifically, given the design intent (i.e., the available struts *i* ∈ 1..*N*, their initial positions $p_{o}^{i}$, final positions $p_{T}^{i}$, and the agents *j* ∈ 1..*M*), TAMP aims to find a feasible sequence of actions *s*_1: *m*_ and a corresponding path *x* that fulfills the constraints that the environment and the design intent imposes. LGP formulates this as an optimization problem:

(3) $$\begin{array}{c} \min_{m,x,s_{1:m}}\int_{0}^{T}c\left(x\left(t\right),\dot{x}\left(t\right),\ddot{x}\left(t\right)\right)dt,\\ s.t.\;\;\;x\left(0\right)=x_{0}\left(p_{o}\right),\;\;x\left(T\right)=x_{T}\left(p_{T}\right),\\ \forall t\in \left[0,T\right]\;\;\;\;\;g\left(x\left(t\right),\;s_{k\left(t\right)}\right)\leq 0,\\ \;\;\;\;\;\;\;\;\;\;\;\;\;\;\;\;\;\;\;\;\;\;\;\;\;\;g_{SW}\left(x\left(t_{SW}\right),\;s_{k}\left(t_{SW}\right),\;s_{k\left(t_{SW}\right)+1}\right)\leq 0,\\ \;\;\;\;\;\;\;\;\;\;\;\;\;\;\;\;\;\;\;\;\;\;\;\;\;\;s_{1:m}\in S \end{array}$$

where the decision variables are the action sequence, *s*_1: m_, its length *m*, and the continuous path *x* of all agents and objects. The cost function *c* (to reduce path length and maximize smoothness) was minimized. The path was constrained by the struts’ start and terminal positions, *p*_O_ and *p*_T_. Further, it had inequalities *g* throughout the path describing joint limits and collision constraints, and constraints *g*_SW_ that describe the switching constraints at an action. Namely, if *s*_*k*(*t*)_ is the action at time *t* (with *k*(*t*) mapping the time *t* to the action that is executed at that time), *g*_SW_ describes switching constraints at time *t*_SW_ between actions $s_{k(t_{SW})}$ and $s_{k(t_{SW})+1}$ (e.g., if an agent needs to grip a strut). The set of feasible action sequences S is described in the Supplementary Material in more detail.

The optimization problem, Equation 3, is hybrid, solving over discrete decisions *m*, *s*_1: m_ (i.e., which agent grasps which strut) as well as the continuous geometric path *x*. Further, the system's kinematic tree changes with actions, implying time‐varying kinematics constraints on the path *x*. Previous LGP solver specifically had to be extended to account for the inversion of kinematic chains in the kinematic tree, and the respective DOF in the configuration. The multi‐bound tree search from^[^ ^29^ ^]^ as the basis for our solver was used. The Supporting Information describes the used, logical actions predicates and logical constraints on action sequences in more detail.

For a given sequence *s*_1: K_, the remaining path optimization problem was non‐convex, and prone to local optima. To address this, the solver was run in parallel with random initializations, and additionally gave the user the possibility to influence the optimizer by setting the number of restarts (in case of infeasibility), and the range of the state initialization. Finally, the solution (or lack thereof) was returned to the ABM, which then takes the new information into account, and continues planning the design of the artifact to be assembled.

### Perception

The coordination of movements of the physical system was conducted by a central computer running the digital twin. The digital twin collects information on the moving parts of the system using the external visual tracking system and sends commands to the individual robotic actuators in order to adjust for tolerances during critical moments in the construction sequence. Specifically, the external vision systems continuously collected the position and rotation of the robotic actuators. The adjustment of the robotic actuators occurs when they were required to grip or release from the timber struts, as accuracy was essential during these operations. When struts were fixed into the environment, their location and rotation was stored in the ABM, and inform the further design process. A video of the collection of the robotic actuator's position and rotation is given in Movie S2 (Supporting Information).

## Conflict of Interest

The authors declare no conflict of interest.

## Supporting information

Supporting information

Supplemental Movie 1

Supplemental Movie 2

Supplemental Movie 3

## Data Availability

The data that support the findings of this study are available from the corresponding author upon reasonable request.
