# Supplementary material for: Leveraging Building Material as Part of the In‐Plane Robotic Kinematic System for Collective Construction
Source: Adv Sci (Weinh). 2022 Jun 24;9(24):2201524. doi: 10.1002/advs.202201524 (PMC9404414; doi:10.1002/advs.202201524)
Supplement: Supplementary file 1 — Supporting information [file ADVS-9-2201524-s001.pdf]

Supporting Information

Leveraging Building Material as Part of the In-Plane Robotic Kinematic System for Collective Construction

Samuel Leder[1,4†], HyunGyu Kim[2,4†], Özgür Salih Ögüz[3,4], Nicolas Kubail Kalousdian[1,4], Valentin Noah Hartmann[3,4], Achim Menges[1,4], Marc Toussaint[3,4*], Metin Sitti[2,4,5,6*]*

1. Supplementary Text

1.1. Additional Commentary on Related Work

Although the field of collective robotic construction is providing generalized methods that can be applied to the implementation of such systems, the field is still very experimental [2]. Therefore, a major contribution of our system is the utilization of an existing building material and its integration into a collective robotic construction system. Although some research is utilizing architectural building materials, they are not at an architectural scale and do not provide enclosure [30]. Other projects utilize non-architectural, highly customized, or stand-in materials, which although ease constraints on the robotic hardware and planning/ control system, would require more research or are generally unfit for application for building construction. **Table S1** summarizes the materials utilized in some existing CRC systems and the constraints of utilizing them. We, therefore, aim to present a next step into transitioning to practice by enabling such systems to utilize structural materials.

Fiberbots utilizes fiberglass, which has history in architectural construction, and therefore similarly works with a real building material [13]. However, the individual Fiberbots do not collaborate, rather just influence the path that other robots travel digitally. In our system, the robots must work together which introduces significant challenges to all aspects of the project.

Table S1. Materials utilized with the existing collective robotic construction (CRC) systems in the literature

Reference	Material	Limitations/Constraint
[6]	Custom Brick	Non-architectural
[11]	Foam Brick	Stand-in for real bricks
[12]	Custom plastic nodes truss members	Non-architectural
[14]	Custom plastic voxel material	Non-architectural
[15]	Custom plastic voxel material	Non-architectural
[16]	Foam, bags of materials, toothpicks	Non-architectural / Non-structural
[19]	Custom brick block	Non-architectural
[20]	Custom strut	Non-architectural
[22]	Filament	Stand-in for structural fiber materials (i.e.carbon or glass fiber)

1.2. Robotic Actuator Details

In the methods section, we explained the general design of the robotic actuator. This section describes the physical actuator in further detail, specifically about gripping and power supply selection. A list of specifications for the robotic actuator is in the **Table S4**.

1.2.1. Gripper

The gripper is designed to form stable connections to the timber struts, lift the timber struts, as well as fully retract. Although the geometry of the gripper frames slightly differs according to the other functionalities of the side of the robotic actuator, the main gripping mechanism are the same in the upper and lower grippers. The gripper arms are mounted to a shaft which runs through the gripper frame. The arms are actuated by a worm gear (1:25) that is mounted directly to a motor shaft on one side of the shaft and supported for rotation by a spur gear (1:1) on the other side. The gripper arms are connected to paddles, which have different variations for the upper and lower grippers.

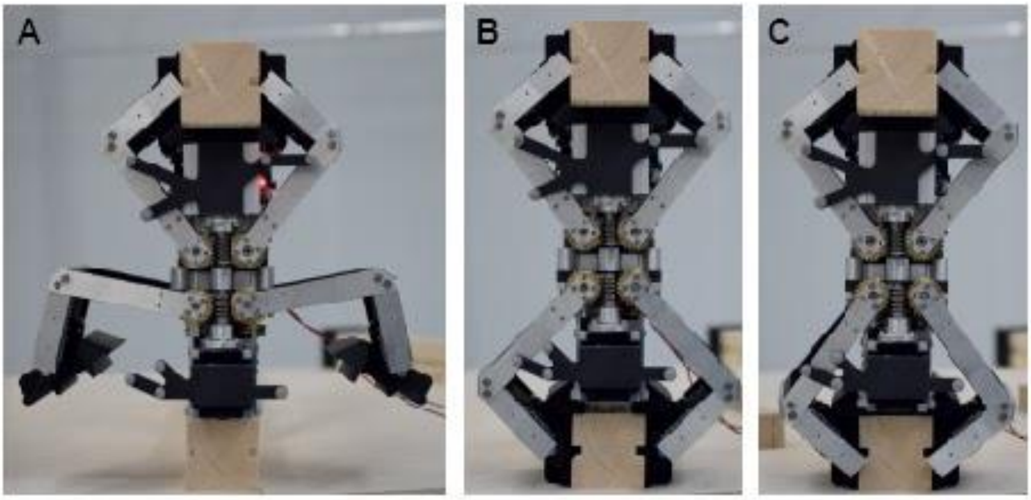

Figure S1. Three main states of the gripper. A single robotic actuator is capable of gripping two timber struts in order to be able to form kinematic chains. The top gripper in the images is lifted. The bottom gripper demonstration of all three possible states of the gripper: (A) full retraction, (B) grasping, and (C) lifting.

Figure S1 indicates the different gripper states: full retraction (**Figure S1**A), grasping (Figure S1B), and lifting (**Figure S1C**). The gripper is open in the full retraction state. To avoid contact when the robotic actuator moves directly above timber struts or building material passes above the robotic actuator, the end-tip of the gripper paddle is higher or lower than the gripping surface of the robotic actuator when the gripper is fully retracted. When the gripper closes, it first enters the grasping state in which the paddles surround the timber strut. Upon further actuation, the gripper enters the lifting state, in which the timber strut is brought closer to the robotic actuator. Videos of all gripper state are given in **movie S3**.

Figure 9E indicates the lifting mechanism. The mechanism includes a linear rail attached to a spring. As such, the length of gripper arms can be shortened and move the gripper paddle inwards toward the robotic actuator to lift the timber strut. In order to achieve both gripping and lifting, the design of the gripper is broken down into three sub-problems: (i) the design of the gripper arms, (ii) the required spring compression, and (iii) the required gripping torque.

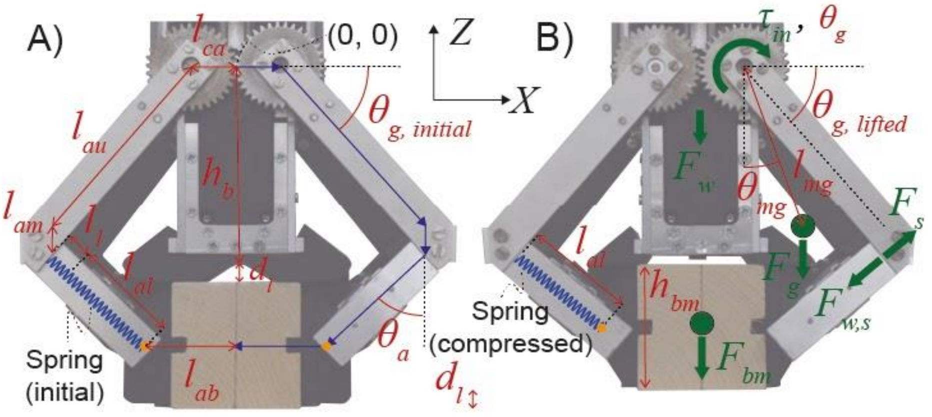

Figure S2. Side-view free-body-diagram of the robotic actuator design. A) Gripper in the grasping state. B) Gripper in the lifting state. Red denotes lengths and angles. Here, l_{ca} is the length between center of body and connecting point of the gripper, l_{au} , l_{am}, l_{al} , and l_l are the lengths of the upper, middle, lower, and changeable parts of the gripper arms, respectively, l_{ab} is the length between the center of timber strut and connecting point of the gripper paddle, l_{mg} is the distance to the center of mass of the gripper from the connecting point of the gripper, d_l is the lifting distance, h_b is the distance between the connecting point of the gripper and bottom of the lower body, θ_g is the angle of gripper, $\theta_{g,initial}$ is the angle at a grasp states of the gripper arm, θ_{mg} is the angle to the center of mass of the gripper, θ_a is the angle of the gripper arm and $\theta_{g,lifted}$ is the angle at a lifted states of the gripper arm]. Green denotes centers of mass, torques and forces [τ_{in} is the torque from the gripper motor, F_g is the weight of a gripper, F_{bm} is the weight of a timber strut, F_s is the force from a spring, F_w is the weight from the kinematic chain, and $F_{w,s}$ is the weight of the kinematic chain for each spring. Blue arrows denote vectors.

In the first sub-problem concerns the lengths, considering compression for lifting, and angles of the gripper arms. These parameters determine how the torque from the gripper motor, τ_{in} , and the weight of the entire robotic actuator, F_w, are transmitted to the gripping force. **Figure S2** depicts free-body diagrams of the gripper configurations for the grasped and lifted states of

the grippers. Detailed specification is listed in **Table S4**. Derived from vector analysis of the free-body diagram, the calculation for the length of the gripper arms is solved using the following equation:

$$V_a\left(\theta_g, l_{au}, l_{am}, l_l\right) = l_{ca}e^{(0)i} + l_{au}e^{(\theta_g)i} + l_{am}e^{\left(\theta_g + \frac{\pi}{4}\right)i} + \left(l_l + l_{al}\right)e^{\left(\theta_g + \frac{\pi}{2}\right)i} + l_{ab}e^{(\pi)i}, \quad \text{(S1)}$$

where l_{ca} is the length between center of body and connecting point of the gripper, l_{au}, l_{am}, l_{al}, and l_l are the length of the upper, middle, lower, and changeable parts of the gripper arms, respectively, and l_{ab} is the length between the center of timber strut and connecting point of the gripper paddle, θ_g is the angle of the gripper.

Solving Equation S1 when the gripper configuration is in the grasped state (**Figure S1**) meaning $\theta_g = \theta_{g,initial} = \theta_a = 45^\circ$, we obtain the following two equations:

$$\begin{aligned} &\text{real}\left(V_a\left(\theta_{g,initial}, l_{au}, l_{am}, l_l\right)\right) = 0, \\ &\text{imaginary}\left(V_a\left(\theta_{g,initial}, l_{au}, l_{am}, l_l\right)\right) = \left(h_b + d_l + 0.5h_b\right)i, \end{aligned} \quad \text{(S2)}$$

where θ_a is the angle of the gripper arm, h_b is the height between the lower gripper's bottom and connecting point of the gripper, d_l is the lifting distance, h_b is the half height of the building material. After lifting (**Figure S1C**) when $\theta_g = \theta_{g,lifted}$, we obtain the following:

$$\begin{aligned} &\text{real}\left(V_a\left(\theta_{g,lifted}, l_{au}, l_{am}, l_l\right)\right) = 0, \\ &\text{imaginary}\left(V_a\left(\theta_{g,lifted}, l_{au}, l_{am}, l_l\right)\right) = \left(h_b + 0.5h_{bm}\right)i, \end{aligned} \quad \text{(S3)}$$

Using Equation S2 and EquationS3, the lengths and angles of the gripper arm, specifically l_{au}, l_{am}, l_l, and $\theta_{g.lifted}$, can be calculated. They are 77.69 mm, 17.23 mm, 13.44 mm, and 50.56°, respectively.

In the second sub-problem, the force required for spring compression is solved. The spring not only helps the lifting mechanism to recover the initial position when the gripping in the fully retracted state, but also decouples the weight of the kinematic chain from the operation

of the lifting mechanism. In the grasping state, the spring undergoes an initial compression in order to remove the effects from the weight of the kinematic chain. The compression force in this state, $F_{w,s}$, is the same as the weight of the kinematic chain divide between the amount of spring which can be calculated using:

$$\begin{aligned} F_w &= N_c\left(m_r + m_{bm}\right)g, \\ F_{w,s} &= \frac{1}{N_s}F_w\cos\left(\theta_a\right) = \mu\delta_{initial}, \end{aligned} \tag{S4}$$

where F_w is the weight from the kinematic chain, N_c is the number of robotic actuators and timber struts in the kinematic chain, m_r and m_{bm} are the mass of the robotic actuator and timber strut, g is the gravitational acceleration, and N_s is the total number of springs in the gripper. We selected the spring coefficient, μ, and initial compressed length, $\delta_{initial}$ in order to satisfy $F_{w,s}$. Before any compression, the length of the spring is $l_{al} + l_l + \delta_{initial}$.

The maximum spring force, $F_{s,Max.}$, and torque to the gripping motor, $\tau_{s,Max.}$, occurs when the gripper is fully compressed in the lifting state, and can be calculated as follows:

$$\begin{aligned} F_{s,Max.} &= \mu\left(l_l + \delta_{initial}\right)e^{\left(\theta_{g,lifted} - \frac{\pi}{2}\right)i}, \\ \tau_{s,Max.} &= \left|\left(l_{au}e^{\left(\theta_{g,lifted}\right)i} + l_{am}e^{\left(\theta_{g,lifted} + \frac{\pi}{4}\right)i}\right) \times F_{s,Max.}\right|, \end{aligned} \tag{S5}$$

Using the information from the two previous sub-problems, we can solve for the static required torque of the gripping motor. There are three kinds of forces applied to a gripping motor, τ_{input}: i) weight of the gripper, F_g, ii) weight of the timber strut, F_{bm}, and iii) force from the spring, F_s. Torque from the weight of the gripper, τ_g, and timber strut, τ_{bm}, is calculated using:

$$\begin{aligned} \tau_g &= l_{mg}\sin\left(\theta_{mg}\right)F_g, \\ \tau_{bm} &= -0.5 l_{ca} F_{bm}, \end{aligned} \tag{S6}$$

where l_{mg} and θ_{mg} are the distance and angle between the connecting point and center of mass of the gripper, respectively. Using this, we can calculate the required torque of the gripping motor, $\tau_{required,g}$, as:

$$\tau_{required,g} = 2\left(\tau_g + \tau_{bm} + 2\tau_{s,Max.}\right)\frac{1}{\eta_g n_g}, \qquad \text{(S7)}$$

where η_g is the efficiency of the motor, and n_g is the gear ratio in the gripping mechanism. The required torque to compress the spring fully is therefore 0.788 *N.m*.

1.2.2. Powering

Although the iteration of the demonstrations was conducted using tethered power, six single lithium polymer batteries (3.7V, 2000 *mAh*) are located in the upper gripper to allow for untethered power. To achieve 11.7 *V*, we connected three batteries serially, and then combined two sets of the three batteries in parallel to increase the battery capacity. The total capacity of the combined battery pack, C_b, is 12 *Ah*. The electronics and motors of the robotic actuator consume 0.3 *A* without any actuation. While each robotic actuator has different power consummations due to slightly different friction in the mechanism, the range of consuming current while rotating is 0.55-0.65 *A*. Therefore the robotic actuator can run for 18.5-21.8 hours if it is continuously rotating. Since the grippers does not need power once they are actuated, they only consume current, C_g, for 10 second at 1.4 *A*. As a result, the total operating time of the robotic actuator would reduce for every time the gripper needs to open and close during its operation. Assuming there is not energy loss, and the robot is continuously rotating with some gripping, the operating time, T_o as follows:

$$T_o = \left(C_b - n_{grip}\left(C_g - C_{ro}\right)\frac{1}{360}\right)\frac{1}{C_{ro}}, \qquad \text{(S8)}$$

where C_{ro} is the averaged current consumption while rotating, and n_{grip} is the number of grips.

1.3. Agent-based model (ABM)

The development of the ABM for generating artefacts designs to be built is based on the ABM framework developed at the Institute for Computation Design and Construction at the University of Stuttgart [31]. The framework is composed of an agent core library to allow for experimentation with agent-based modelling. The core is supplemented by application-specific libraries and expert libraries, which enable in depth explorations of specific application domains. This works contributes to the ABM framework by beginning to establish a collective robotic construction application-specific library. The aim of the library is to introduce classes that allow for experimentation with the framework on research related to collective robotic construction systems.

The major development of the collective robotic library associated to this work is the introduction of a class which helps to define the agent as a mobile robot. As compared to the plate system reported on in [31], the agent~~,~~ in the ABM developed for this work~~,~~ is a representation of the robotic system rather than the material system. Therefore, a class that helps to describe a mobile robot was introduced. This includes parameters to define the working time and kinematic reach of the robot.

Using this new agent definition, the agent is defined by a kinematic chain in this work. At each iteration of the model, the behaviors of the agents (seek, orient, and cull) negotiate to reveal a proposed strut or struts to be built into the environment. As pervious discussed, the behaviors are influenced by the design intent parameters in **Table 1**. By updating the design intent parameters and running the ABM, highly varied artefacts can be designed (**Figure S3**). This showcases the flexibility of the system to be able to build structures of varied spatial quality according to user preference. In the future, this could be adapted to address further design criteria such as structural load or environmental conditions.

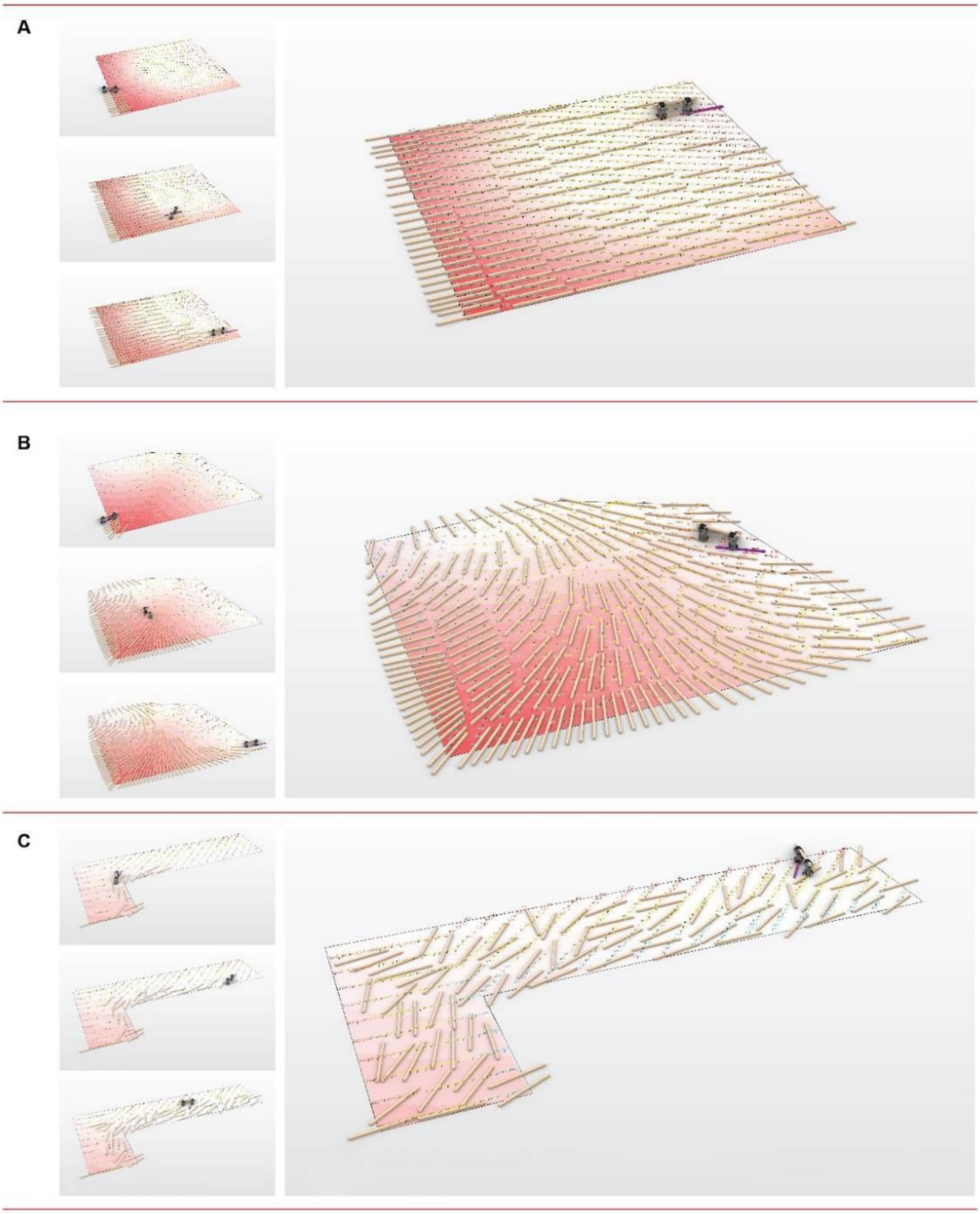

Figure S3. Design variation achieved with the agent-based model. Using the design intent parameters of orientation field, placement priority, density, strut supply and bounding area for the ABM, highly differentiated designs can be iteratively created. The smaller images are snapshots from the steps before the larger image, indicating the process of construction. (A) Rectilinear boundary, constant orientations, gradient density. (B) Polygon boundary, aligned orientations, gradient density. (C) L-shaped boundary, random orientation, low density variation.

1.3. Planning

Our planner builds on a state-of-the-art task and motion planning framework, namely Logic-Geometric Programming (LGP). Due to the challenges of target tasks in this work, new skills and specific modifications have been introduced to the original LGP formulation. There are five high-level actions/skills: walk, chain, pick, handover, and place. Each of those actions requires updating the kinematic chain formed by the robot-material sequences accordingly. This dynamic nature of the robotic structure imposes challenges for both task and motion planning components. For instance, consider the locomotion task with the kinematic chain formed by a $\left[\mathrm{strut_i}\text{-}\mathrm{robot_m}\text{-}\mathrm{strut_j}\text{-}\mathrm{robot_n}\right]$ sequence: for the first step action while one end of this chain, $\left(strut_i - robot_m\right)$ pair, acts as a support, and the other end, $robot_n$, as an end-effector, for the following step the order has to change. In essence, high-level action planner must be capable of updating the logical states of the world accordingly, and similarly the motion planner must be capable of updating the kinematic chain to properly optimize for feasible motion paths. By introducing special logical predicates that inform our planner to update the kinematic tree of the current configuration whenever necessary, the nonlinear program solver optimizes the motion path to satisfy kinematic and geometrical constraints properly (**Table S2**).

Here, we provide each action for completeness, and highlight the critical predicates (functionalities). These actions, together with the predicates make up the set of possible sequences

$$\mathbb{S} : \left\{ \left\{ s0, \ldots, s_m \right\} \middle| s_{\mathbf{i+1}} \in succ\left(s_i, a_i\right) \forall i \right\}. \tag{S9}$$

with a_i being the mode switch, s_i being the action, and the *succ*() operator describing the actions that are feasible follow-up actions to action s_i, which is imposed by the predicates (**Table S3**) that have to be fulfilled for the following action.

Table S2. Decision rules and the motion constraints that they imply

Action	Optimization predicates
(walk A1 A2 S1 S2)	[stable S2 A2]! [stable S1 A1] [attached S1 A1]
(chain A1 A2 B1 B2 S1 S2 S3)	[stable A2 S2]! [stable B2 S2] [attached S2 B2]
(pick A1 A2 S1 S2)	[stable A1 S1] [attached S1 A1]
(handover A1 A2 B1 B2 S1 S2 S3)	[stable A1 S1]! [stable B1 S1] [attached S1 A1]! [attached S1 B1]
(place A1 A2 S1 S2 G)	[stable A1 S1]! [stable G S1] [above S1 G]

Predicate	Constraints
[Stable X Y]	create a stable joint from X to Y, constrained to velocity 0
[above X Y]	X is above Y with a distance of > 0
[attached X Y]	(aboveStrut) relative position of X (endeffector) w.r.t. Y (strut) in x-axis is 0, and in y-axis stays within the boundaries of the strut, (scalarProductXY) the x-axis of X (endeffector) is perpendicular to the y-axis of Y (strut)

Table S3. Predicates used in the path optimization as the constraints

Table S4. Specifications of the experimental system

**Robotic actuator**	
Overall dimension	138 (depth) x 179 (wide) x 309.5 (height) mm^3
Weight	2.8 *kg* (m_r)
Degree of freedom	1 (continuous rotating)
Number of actuators	3 (MX-106R, DYNAMIXEL, ROBOTIS) : rotating, lower, and upper grippers. Efficiency of the actuator ($\eta_{rotation}, \eta_{gripping}$): 49%
Battery	6-cell LiPo batteries (3.7 *V*, 2000 mAh)
Communication	Bleutooth (BT-410, ROBOTIS)
Material	Body frame: 3D printed carbon chopped plastic material<br>Gear: brass and steel<br>Shaft: titanium
Gear ratio for the rotation ($n_{rotating}$)	38.46:1 (25:1 worm gear connection, and 40:26 spur gear connection)
Gear ratio for the gripping ($n_{gripping}$)	25:1 (25:1 from a worm gear connection)
Processor	OpenCM 9.04 with 485EXP, ROBOTIS
Gripper dimension (Figure S2)	l_{ca} = 17 mm, l_{au} = 77.69 mm, l_{am} = 17.23 mm, l_l = 13.44 mm, l_{al} = 34.56 mm, l_{ab} = 38 mm, $\theta_{g,initial}$ = 45 degree, $\theta_{g,lifted}$ = 50.56 degree, θ_a = 45 degree, l_{mg} = 82.31 mm, θ_{mg} = 25.66 degree, m_g = 0.212 *kg*, h_b = 74.1 mm
Stiffness of the spring in the lifting mechanism	1.08 *N*/mm (μ) with initial compressed length of 59.811 mm ($\delta_{initial}$)
Lifting height (d_l)	7 mm
**Timber strut**	
Density	$883.2\,Kg/m^3$
Cross section (h_{bm})	50 (wide) x 50 (height) mm with groove (6 x 6 mm)
Weight (m_{bm})	$\text{Density} \times h_{bm} \times h_{bm} \times \text{length of the strut}$

Supplementary Movies

Movie S1. Videos of five experimental demonstrations. A compilation of five videos, each describes one of the five demonstrations discussed in the paper (Demonstration I-V). The videos appear in the same order as discussed in the paper, the three examples of locomotion, followed by dynamic kinematic chains, and ending with transportation.

Movie S2. Close-up video of the rotation correction. Close-up video of the kinematic chain of two robotic actuators connected by a timber strut rotating in order to pick up a timber strut in the environment. The kinematic chain first rotates to the strut it aims to grip, corrects it position, and then picks up the strut.

Movie S3. Close-up video of three gripping states. Close-up video displaying the states of possible three states of the grippers of the robotic actuators. The gripper in the video started fully retracted, then grasps a timber strut, and finally lifts it.
